# Inverse association between gastroesophageal reflux and blood pressure: Results of a large community based study

**DOI:** 10.1186/1471-230X-8-10

**Published:** 2008-04-15

**Authors:** Liam J Murray, Peter McCarron, Roger B McCorry, Lesley A Anderson, Athene J Lane, Brian T Johnston, George Davey Smith, Richard F Harvey

**Affiliations:** 1 Northern Ireland Cancer Registry, Department of Epidemiology and Public Health, The Queens University of Belfast, Belfast, UK; 2 Bolton Hospitals NHS Trust, Bolton, UK; 3 Department of Social Medicine, University of Bristol, Bristol, UK; 4 Royal Hospitals Trust, Belfast, UK; 5 Frenchay Hospital, North Bristol NHS Trust, Bristol, UK

## Abstract

**Background:**

In a cross-sectional community based study, as part of a randomised controlled trial of eradication of Helicobacter pylori infection, the association between blood pressure and symptoms of gastro-oesophageal reflux was examined.

**Methods:**

Linear regression was used to examine the association between systolic and diastolic blood pressure and the frequency of heartburn and acid regurgitation in 4,902 of 10,537 participants aged 20–59 years.

**Results:**

In multivariable analyses, adjusted mean systolic blood pressure was 4.2 (95% confidence interval 1.5 to 7.0) mm Hg lower in participants with daily acid regurgitation compared to those with less frequent symptoms. Similarly, for diastolic blood pressure, a reduction of 2.1 (0.0 to 4.3) mm Hg wasobserved.

**Conclusion:**

People who experience daily symptoms of gastro-oesophageal reflux have lower blood pressure than people with less frequent or no symptoms. It is possible that factors influencing nitric oxide concentrations both at the lower oesophageal sphincter and within the vasculature may be involved. This hypothesis requires confirmation.

**Trials registration number:**

ISRCTN44816925

## Background

In 2003 we reported reduced stroke mortality among patients with oesophageal columnar epithelium (Barrett's oesophagus), with cerebrovascular deaths in patients with specialised intestinal metaplasia of the oesophagus being half that of the general population [1]. Subsequently, a postmarketing surveillance study of 18,000 patients on omeprazole in the United Kingdom has not shown any reduction in stroke mortality in these patients compared to the general population [2] but this drug is often prescribed for upper gastrointestinal conditions other than gastro-oesophageal reflux and Barrett's oesophagus. We hypothesised that the association we observed may be due to individuals with reduced lower oesophageal sphincter (LOS) pressure, (a risk factor for gastro-oesophageal reflux and Barrett's oesophagus) also having low vascular tone and blood pressure, resulting in reduced stroke risk. To investigate this, we examined whether blood pressure is associated with symptoms of gastro-oesophageal reflux.

## Methods

From 1996 to 1998, 10,537 individuals, aged 20–59 years, registered with seven general practices in Southwest England were enrolled in a community based randomised controlled trial of eradication of *Helicobacter pylori (H. pylori)*. The trial obtained ethics approval from Frenchay Hospital LREC (reference number: 95/83 20/01/1996) and participants provided written informed consent. Participants provided information on the frequency of dyspeptic symptoms experienced in the previous three months, including heartburn ("a burning or ache behind the sternum not due to heart trouble") and acid regurgitation ("a very sour or acid tasting fluid at the back of the throat") using a validated questionnaire [3]. In addition, blood pressure, height, and weight were measured and data on antihypertensive medication, smoking history, alcohol and coffee consumption, and adult social class (derived from current occupation) were collected. The current analysis is based on all participants who tested positive for *H. pylori *infection (n = 1,634) and a computer generated random sample of *H. pylori *negative participants (n = 3,268) to give a *H. pylori *negative to positive ratio of 2:1

Linear regression was used to examine the association between systolic and diastolic blood pressure and the frequency of heartburn and acid regurgitation (daily vs. less frequent). Analyses took account of the clustered nature of the data (by general practice), and the increased sampling of *H. pylori *positive participants – 33% of participants included in the database, but 15.5% of all participants screened – was dealt with by applying weights that were proportional to the inverse of the sampling fractions. Initial analyses were adjusted for age (years) and sex, while in fully-adjusted analyses we additionally controlled for body mass index (weight(kg)/height^2^(m), smoking habit (never; ex-smoker; current smoker, <20 a day; current smoker = 20 a day), weekly alcohol intake in units (none; <10; 10–19; = 20), daily coffee consumption in cups (none; 1–4; = 5) and adult social class (manual, non-manual). Similar analyses were conducted to examine whether the frequency of the occurrence of other dyspeptic symptoms (epigastric pain/discomfort, nausea, wind and belching) was associated with blood pressure. Since age-adjusted results were similar for the total sample and the 4,227 (86.2%) individuals with data available on all confounders we report only the latter here. Analyses were carried out using Stata 7.0 (College Station, Texas, USA).

## Results

One hundred and seven (2.5%) and 66 (1.6%) of the 4,227 participants with complete data experienced daily heartburn or acid regurgitation respectively. Participants experiencing either daily acid regurgitation or heartburn symptoms were heavier, and smoked more cigarettes than people with less frequent symptoms, while those with daily heartburn consumed more alcohol and were more likely to come from manual social class (Table 1). In age and sex-adjusted analyses, restricted to individuals not receiving antihypertensive medication (258 excluded), participants with daily acid regurgitation had lower mean systolic and diastolic blood pressure than individuals who had less frequent or no acid regurgitation (Table 2). Controlling for potential confounders strengthened these associations. There was no association between daily heartburn or the other dyspeptic symptoms and blood pressure.

**Table 1:** Characteristics of participants with and without acid regurgitation or heartburn

	Acid regurgitation, n (%)		Heartburn, n (%)	
	No	Yes	No	Yes
All participants	4161 (98.4)	66 (1.6)	4120 (97.5)	107 (2.5)
Males	1985 (47.7)	23 (36.5)	1952 (47.4)	57 (53.3)
Mean age (SD)	45.6 (8.9)	45.4 (9.3)	45.5 (9.0)	47.5 (8.5)
Receiving antihypertensives	255 (6.1)	3 (4.6)	255 (6.1)	3 (2.8)
Mean BMI (SD)	26.7 (4.4)	28.8 (4.6)	26.8 (4.4)	28.9 (5.2)
Cigarettes				
Never	2204 (53.0)	31 (47.0)	2183 (53.0)	52 (48.6)
Ever	987 (23.7)	8 (12.1)	976 (23.7)	19 (17.8)
Current, <20/day	597 (14.4)	8 (12.1)	589 (14.3)	16 (15.0)
Current, = 20/day	373 (9.0)	19 (28.8)	372 (9.0)	20 (18.7)
Alcohol (units/wk)				
None	659 (15.8)	20 (30.3)	651 (15.8)	28 (26.2)
1–9	1966 (47.2)	26 (39.4)	1955 (47.4)	37 (34.6)
10–19	883 (21.2)	10 (15.2)	877 (21.3)	16 (15.0)
≥ 20	653 (15.7)	10 (15.2)	637 (15.5)	26 (24.3)
Coffee (cups/day)				
None	801 (19.2)	20 (30.3)	792 (19.2)	29 (27.1)
1–4	2209 (53.1)	28 (42.4)	2182 (53.0)	55 (51.4)
≥ 5	1151 (27.7)	18 (27.3)	1146 (27.8)	23 (21.5)
Non- manual social class	2667 (64.1)	44 (66.7)	2653 (64.4)	58 (54.2)
Mean systolic BP (SD)	119.8 (21.7)	119.2 (19.4)	119.8 (21.8)	121.0 (16.2)
Mean diastolic BP (SD)	75.6 (19.2)	75.1 (10.6)	75.6 (19.3)	76.4 (12.0)

Data shown relate to 4227 participants with complete data

**Table 2:** Association between blood pressure and frequency of acid regurgitation and heartburn

Symptom, frequency	n^1^	Mean difference in systolic blood pressure (mm Hg) (95% CI), p		Mean difference in diastolic blood pressure (mm Hg) (95% CI), p	
		Age and sex adjusted	Fully-adjusted^2^	Age and sex adjusted	Fully-adjusted^2^
Acid regurgitation					
Daily	63	-2.5 (-4.2 to -0.8), 0.012	-4.2 (-7.0 to -1.5), 0.009	-0.9 (-2.9 to 1.2), 0.34	-2.1 (-4.3 to 0.0), 0.053
Less frequently	3906	1.00 (reference group)	1.00	1.00	1.00
					
Heartburn					
Daily	104	1.7 (-3.7 to 7.1), 0.48	-0.5 (-6.8 to 5.8), 0.85	-0.6 (-1.3 to 2.5), 0.46	-0.9 (-2.8 to 1.1), 0.32
Less frequently	3865	1.00	1.00	1.00	1.00

^1 ^Number of participants for whom full data available

^2 ^Adjustment for age, sex, BMI, antihypertensive medication, smoking, coffee and alcohol intake, and social class

## Discussion

We found an inverse association between experiencing daily acid regurgitation and blood pressure. This was a large, population based study, whose participants were representative of the total sample. A wide age range of both sexes were studied producing novel findings. Weaknesses, however, include the fact the study was symptom based with no direct information regarding the underlying endoscopic diagnosis. Some researchers suggest that for heartburn a more complete 'word picture' than we used is required to avoid misreporting this symptom [4]. Misclassification may therefore have contributed to the weak relationship seen between blood pressure and heartburn. Moreover, as few participants in our study had daily reflux symptoms, our results need to be viewed as preliminary. However, there are several emerging strands of evidence to support a causal relationship between gastro-oesophageal reflux and low blood pressure. Firstly, this finding is consistent with our previous observation of lower stroke mortality in patients with Barrett's oesophagus in a separate cohort from Northern Ireland [1]. Secondly, patients with gastro-oesophageal reflux disease have been shown to have decreased sympathetic function, with blunting of blood pressure responses to stress [5]. Thirdly, recent research into nitric oxide (NO) provides a mechanistic link between gastro-oesophageal reflux and low blood pressure. NO functions as the major non-adrenergic non-cholinergic neurotransmitter in the autonomic nervous system [6] and NO-mediated action appears to be crucial in governing LOS pressure [7]. Patients with gastro-oesophageal reflux have elevated levels of serum nitrate compared to controls [8] and infusion of a NO synthase blocker has been seen to induce both raised resting LOS tone and, in keeping with the well known vasodilatory effects of NO, raised blood pressure [9].

## Conclusion

We suggest that factors influencing the endogenous production of NO underlie the observed association between symptoms of gastro-oesophageal reflux and blood pressure. Confirmation of these findings, and further investigation of the pathophysiological role that NO may play in gastro-oesophageal reflux, and possibly Barrett's oesophagus, are warranted.

## Competing interests

The author(s) declare that they have no competing interests.

## Authors' contributions

LM, and RH were involved in planning and obtaining funding for the Bristol Helicobacter project. JAL managed the project. LM proposed the hypothesis for this paper. LM and PMcC carried out the analyses and wrote the initial draft of the paper. All the authors commented critically on the initial draft, and contributed to the final version of the paper. LM is the guarantor.

## Pre-publication history

The pre-publication history for this paper can be accessed here:


